# Supplementary material for: Facilitators and barriers to colorectal cancer screening using the immunochemical faecal occult blood test among an average-risk population in semi-rural Malaysia: A qualitative study
Source: PLoS One. 2022 Dec 29;17(12):e0279489. doi: 10.1371/journal.pone.0279489 (PMC9799312; doi:10.1371/journal.pone.0279489)
Supplement: S1 Checklist — (DOCX) [file pone.0279489.s001.docx]

**Manuscript: *Facilitators and barriers to colorectal cancer screening using the immunochemical faecal occult blood test among an average-risk population in semi-rural Malaysia: a qualitative study***

**Consolidated criteria for reporting qualitative studies (COREQ): 32-item checklist**

Developed from:

Tong A, Sainsbury P, Craig J. Consolidated criteria for reporting qualitative research (COREQ): a 32-item checklist for interviews and focus groups. *International Journal for Quality in Health Care*. 2007. Volume 19, Number 6: pp. 349 – 357

| **No. Item** | **Guide questions/description** | **Reported on Page #** |
| --- | --- | --- |
| **Domain 1: Research team and reﬂexivity** |  |  |
| *Personal Characteristics* |  |  |
| 1. Inter viewer/facilitator | Which author/s conducted the interview or focus group? | Line 107 |
| 2. Credentials | What were the researcher’s credentials? E.g., PhD, MD | Line 107 |
| 3. Occupation | What was their occupation at the time of the study? | **The researcher was a PhD candidate at the time of the study.** |
| 4. Gender | Was the researcher male or female? | Line 107 |
| 5. Experience and training | What experience or training did the researcher have? | Line 107 |
| *Relationship with participants* |  |  |
| 6. Relationship established | Was a relationship established prior to study commencement? | **Yes –** Line 98-99, 102-106 |
| 7. Participant knowledge of the interviewer | What did the participants know about the researcher? e.g., personal goals, reasons for doing the research | **Participants were briefed on the purpose of the study and understood it. Ethical had granted, participants reviewed the participant information documentation prior to giving their written informed consent to be involved.** |
| 8. Interviewer characteristics | What characteristics were reported about the inter viewer/facilitator? e.g., Bias, assumptions, reasons and interests in the research topic | **Not reported in the manuscript. The interviewer (KR) was the full-time PhD candidate working on this study.** |

| **Domain 2: study design** |  |  |
| --- | --- | --- |
| *Theoretical framework* |  |  |
| 9. Methodological orientation and Theory | What methodological orientation was stated to underpin the study? e.g., grounded theory, discourse analysis, ethnography, phenomenology, content analysis | Line 115-126 |
| *Participant selection* |  |  |
| 10. Sampling | How were participants selected? e.g., purposive, convenience, consecutive, snowball | Line 98-99 |
| 11. Method of approach | How were participants approached? e.g., face-to-face, telephone, mail, email | **Participants approached face-to-face.** |
| 12. Sample size | How many participants were in the study? | Line 156 |
| 13. non-participation | How many people refused to participate or dropped out? Reasons? | **None** |
| *Setting* |  |  |
| 14. Setting of data collection | Where was the data collected? e.g., home, clinic, workplace | **Public community center** |
| 15. Presence of non-participants | Was anyone else present besides the participants and researchers? | **SEACO field staff & a professional Chinese translator** |
| 16. Description of sample | What are the important characteristics of the sample? e.g., demographic data, date | Page 7 Table 1 |
| *Data collection* |  |  |
| 17. Interview guide | Were questions, prompts, guides provided by the authors? Was it pilot tested? | (Line 126 - 130) |
| 18. Repeat interviews | Were repeat inter views carried out? If yes, how many? | **No** |
| 19. Audio/visual recording | Did the research use audio or visual recording to collect the data? | Line 112 |
| 20. Field notes | Were ﬁeld notes made during and/or after the interview or focus group? | Line 140 |
| 21. Duration | What was the duration of the inter views or focus group? | Line 108 |
| 22. Data saturation | Was data saturation discussed? | Line 150-154 |
| 23. Transcripts returned | Were transcripts returned to participants for comment and/or correction? | **No. The findings were presented during Community Engagement Committee’s annual meeting which was held online on the 31^st^ March 2022.** |
| **Domain 3: analysis and ﬁndings** |  |  |
| *Data analysis* |  |  |
| 24. Number of data coders | How many data coders coded the data? | Line 140-141  **Two coders** |
| 25. Description of the coding tree | Did authors provide a description of the coding tree? | Line 141-150 |
| 26. Derivation of themes | Were themes identiﬁed in advance or derived from the data? | **Themes were derived from the data** |
| 27. Software | What software, if applicable, was used to manage the data? | **NVivo version 12.0.** |
| 28. Participant checking | Did participants provide feedback on the ﬁndings? | **No** |
| *Reporting* |  |  |
| 29. Quotations presented | Were participant quotations presented to illustrate the themes/ﬁndings? Was each quotation identiﬁed? e.g., participant number | Line 173-334  **Yes** |
| 30. Data and ﬁndings consistent | Was there consistency between the data presented and the ﬁndings? | Table 2, Page 8  **Yes** |
| 31. Clarity of major themes | Were major themes clearly presented in the ﬁndings? | Line 173-334,  Table 2, Page 8  **Yes** |
| 32. Clarity of minor themes | Is there a description of diverse cases or discussion of minor themes? | **Yes, attached as S2 Appendix** |
