# Supplementary material for: Facilitators and barriers to colorectal cancer screening using the immunochemical faecal occult blood test among an average-risk population in semi-rural Malaysia: A qualitative study
Source: PLoS One. 2022 Dec 29;17(12):e0279489. doi: 10.1371/journal.pone.0279489 (PMC9799312; doi:10.1371/journal.pone.0279489)
Supplement: S1 Appendix — (DOCX) [file pone.0279489.s002.docx]

| **Briefing** |
| --- |
| - Introduce yourself and clarify the purpose of the interview with the participants. - Explain the length and structure of the interview as well as the requirement to note-take and record (with the consent of the participant). - Assure that there are no right or wrong answers as well as the confidentiality of the interview stating that the participants will remain anonymous and will not be identifiable. - Ask participants if they have any questions before proceeding with the interview. |
| **Aim** |
| - To elicit views about the barriers and enabling and facilitating factors to, and the ‘costs’ and benefits of, participating in population-based colorectal cancer screening. |
| **Objectives** |
| Explore and facilitate discussion regarding:   - health check-ups and use of preventative health services. - colorectal cancer. - colorectal cancer screening. - opportunistic colorectal cancer screening - population-based colorectal cancer screening. - susceptibility, barriers, benefits and self-efficacy towards colorectal cancer screening. - enablers to participate in population-based colorectal cancer screening. |
| **Questions**  **i) Health check-ups** |
| 1. What do you usually do when you are sick or have concerns about your health?   *Probe:* seek information online/ call helpline, e.g. NGO/ speak to family or friends/ buy medication in pharmacies/ see alternative specialist/ see doctor   1. Is it better to know about your risk of developing a disease? Why? 2. Would you prefer not to know about it until it happens? Why? 3. If there was a test for (any) disease, would you want to take it? Why/ Why not? 4. What is your experience with health check-ups? (eg, do you see the doctor for blood pressure check-ups, diabetes check-ups or blood tests)?   *Probe:* Has your doctor ever recommended any health check-ups/ screens/ blood test?  *Probe:* Have you ever had a health check-up/ screening/ blood test without your doctor recommending it? If yes, why? If no, why not? |
| **ii) Awareness about cancer and cancer screening** |
| 1. What cancer signs and symptoms are you aware of? 2. What would you do if you think you experienced symptoms that might be signs for cancer?   *Probe:* Who would you talk to about the symptoms you’re experiencing?   1. Can you describe the experiences you have had with any type of cancer screening?   *Probe*: How do you feel about attending cancer screening (in general)?/ Did anyone ever recommend you to go for cancer screening? |
| **iii) Colorectal cancer & screening** |
| 1. What do you know about colorectal cancer?   *Probe:* Signs/ symptoms/ who is at risk/ how severe is the disease/ personal susceptibility   1. What do you know about colorectal cancer screening?   *Probe:* Are you aware that you can be screened for colorectal cancer?  If yes, what does the screening involve?  If no, what do you think is involved?   1. Can you describe the experiences you have had with colorectal cancer screening?   *Probe:* If participant had experience with CRC screening, - how do you feel about attending colorectal cancer screening?  *Probe:* Has anyone recommended colorectal cancer screening to you in the past?  *Probe:* Did you ever discuss colorectal cancer screening with anyone?  *Interviewer explains the procedure of the iFOBT (stool test)*   1. How do you feel about the procedure as we have described it?   *Probe:* What do you think are the positive things about the screening? (*e.g.* health benefits, early detection, better chances of positive outcome, less severe treatment & cost saving)  *Probe:* What do you think are the negative things about the screening? |
| **iv) Colorectal cancer screening invitation** |
| *Interviewer explains the current Ministry of Health opportunistic screening programme.*   1. How would you feel if your doctor asked you to take a colorectal cancer screening test? 2. How would you feel if the invitation for the colorectal cancer screening test came with the post? What would you do? (even though the doctor never mentioned it to you) 3. Colorectal cancer risk increases with age. In the UK, everyone aged 50 and above receives an invitation to attend colorectal cancer screening every three years. How would you feel about completing a colorectal cancer screening even though you don’t have any symptoms? 4. What would be the best way for you to receive the invitation/ test kit?   *Probe:* Post/ collect from clinic  *Probe:* How do you receive post?  *Probe:* who should that invitation come from (health clinics – government/ private; NGO; ministry of health, university, SEACO, others? |
| **v) Barriers towards colorectal cancer screening** |
| 1. Are there any issues/ concerns that would hold you back from completing colorectal cancer screening?   *Probe:* Not knowing what it is about/ Not necessary (due to no symptoms/ not at risk)/ Cost / Fear of procedure and/or cancer/ Time/ Doctor did not recommend it/ Distance to travel/ Feeling of embarrassment/ Practicality of sending stool/ Dislikes idea of taking stool sample/ preference usually go to private clinic – don’t want to go to government clinic/ screening is painful/ concerns about hygiene  *Probe:* How do you feel to collect stool at home and bring it to the clinic? |
| **vi) Enabling and facilitating factors to attend colorectal cancer screening** |
| 1. What would encourage you/ your friends/ family members to take a colorectal cancer stool test? / What would make it easier for you to participate?   *Probe:* Education/ more knowledge about procedure/ information about colorectal cancer (what kind of information?)/ other people recommending it (who?) / cost/ transport/ sending test through post/ explanation of how to take stool sample and send to clinic  *Probe:* Do you think receiving reminders to complete the tests would encourage you to complete it? If yes, what kind of reminders (text message, letter, phone call, email, etc)? |
| **vii) Receiving test results** |
| 1. How do you feel about getting the test results?   *Probe:* relief/ worry/ shame   1. What would be the best way to follow up to inform you about the screening result? |
| **Conclusion** |
| - Draw conclusions from the discussion and ask for any further comments - Thank participants for their participation |
