# Supplementary material for: Facilitators and barriers to colorectal cancer screening using the immunochemical faecal occult blood test among an average-risk population in semi-rural Malaysia: A qualitative study
Source: PLoS One. 2022 Dec 29;17(12):e0279489. doi: 10.1371/journal.pone.0279489 (PMC9799312; doi:10.1371/journal.pone.0279489)
Supplement: S2 Appendix — (DOCX) [file pone.0279489.s003.docx]

| **Key-themes** | **Sub-themes** | **Quotes** |
| --- | --- | --- |
| Cancer awareness | Limited awareness about CRC signs/ symptoms and screening | *I have heard from radio saying that a lot of people suffer from this cancer, so it is better to do a screening test. But I am not so sure about the test.* ***(Indian/F)***  *I've heard of it but don’t quite understand this colon cancer.* ***(Malay/F)***  *I've heard of it, too but not the signs.* ***(Indigenous/M)***  *I just hear it like that, in a casual manner. I don’t know in detail.* ***(Indigenous /F)***  *I feel this test could protect us and early detection might help in early treatment. But so far, no one has told me about this test.* ***(Indian/F)***  *I've heard of the stool test, but I don't know how to do it.* ***(Indigenous /M)***  *It is like putting a pipe inside, scope procedure. Blood test, it will let us know the index of the cancer. But we are not aware of the stool test.* ***(Chinese/F)***  *No, it is my first-time hearing of the stool test.* ***(Chinese/M)*** |
|  | CRC awareness through affected family/ friends | *My father has colorectal cancer. In the last phase of cancer, he had stomach pain and could not defecate.* ***(Chinese/M)***  *My uncle had colon cancer, as well. I took him to do an X-ray and scan at a private hospital. The X-ray wasn't clear. But the scanning was precise, and the doctor showed to me. It was too late for treatment. (****Indigenous /M)***  *When someone has a family member or friend that experiences cancer, then only will be more concerned to do anything related to it. Otherwise, people will ignore it.* ***(Indian/M)***  *Yes, my mom has colon cancer. So initially she had a sort of stomach-ache, then the stool was dark colour. So, I just went to see the doctor, checked and confirmed it was colon cancer. So, I think that would be the initial symptoms.* ***(Chinese/F)***  *There are a few symptoms that I am aware of about colorectal cancer. For instance, pain stomach-ache, incomplete defecation, pain during defecation and blood in the stool.* ***(Indian/M)***  *Stool looks black in colour, stool is smaller than usual, loss of weight, no sign of pain even the body part has cancer, lack of appetite.* ***(Chinese/M)***  *My friend did get colorectal cancer. By the time they got to know about it, it was already late stage. When they checked, doctor detected bloody stool and had constipation. Then, doctor advised to undergo surgery to remove the intestine.* ***(Malay/F)*** |
|  | Perceived severity of late-stage cancers (general) | *My wife had breast cancer. She complained of pain then I brought her to the hospital, but it was too late. She is already in stage 4.* ***(Indian /Male)***  *My sister had experienced massive bleeding during this period, went for a few check-ups but did not progress well as she was in stage 4 of cervical cancer.* ***(Malay/F)***  *I relate a case of my brother. He has pancreatic cancer; he could not digest well, feel like not to eat anything. He went for a check and thought it is stomach problem. Then after the MRI test, the result shows the pancreatic has a small tumour. He has a lot of tumours inside, so the doctor said he could not be operated.* ***(Chinese/F)***  *I feel so scared when I hear about cancer. It is better to protect early by doing the test.* ***(Indian/F)***  *Yes, yes. If we knew about cancer, if late, then no chance, one month then it is done.* ***(Chinese/M)*** |
| Health-seeking behaviour | Self-medication for minor illnesses | *It's like this, we will take our own health supplement or medicine at first, see what the symptoms are, then we will take what kind of medicine la.* ***(Chinese/F)***  *So, I think I can overcome it myself. I know what sickness I'm experiencing. I mean, like if it’s a joint ache; I’d consume some medicine.* ***(Malay/M)***  *A little bit of feeling sick, then I will take Panadol, along that will take vitamin. Just for a shorter period, I will take vitamin C.* ***(Indian/F)***  *Yes, we could just go to the pharmacist at the counter, tell them what’s wrong, they give the medication, we pay and leave instead of waiting for hours sometimes at the clinic for the turn for consultation.* ***(Indigenous /M)*** |
|  | Trust in doctor | *I will do it when the doctor asks. Definitely will ask the doctor, reason for doing the test, without proper explanation it would be doubtful to do the test. I feel it is not necessary to do, as I am alright*. ***(Indian/M)***  *Accept, if the doctor mentions it, I will. It is because if the doctor did not mention it, we will not know about it.* ***(Chinese/F)***  *We have to accept what the doctor is saying and ask us to do. Doctors usually will tell us something which is for our own benefits. Therefore, we have to follow the doctor's advice on doing the test.* ***(Indian/F)***  *I’d be happier still if the stool test offered to me.* ***(Malay/F)*** |
|  | Willingness to participate in secondary prevention, monitoring and management of health conditions | *Yes, I have done it once. I went to this medical camp organised by the Hindu Sangam and did cancer screening tests, including stool test for colon cancer. They said no problem. I did about 1 to 2 months ago and will repeat the test as recommended.* ***(Indian/M)***  *The minute we know the symptoms, we should get treatment for it. Don't wait, ahh. No need to wait.* ***(Malay/F)***  *Yes, I will go for a medical check-up by myself. The most important is we should not let our body stressed, just go for a medical check-up.* ***(Chinese/M)*** |
| Motivation to participate in iFOBT screening | Self- efficacy to participate in screening and collect stool | *I don’t see any difficulties doing the stool test.* ***(Indian/M)***  *This is one of the ways to protect ourselves from the bowel cancer. If we do the stool test, it would be helpful to get to know the risk for the cancer.* ***(Indian/F)***  *I can do the stool test as this is my chance to know better about the bowel cancer.* ***(Malay/M)***  *For early detection, I will do the stool test, at least I know my condition through the stool test.* ***(Indigenous /M)***  *The stool test is simple and I’m willing to do it.* ***(Chinese/M)***  *The stool test is convenient and easy to do it.* ***(Chinese/F)***  *I want to know my condition, so must do it. Disgusted or not, nauseated or not, I can and will do the stool test.* ***(Malay/F)*** |
|  | Perceived benefits of CRC screening and early detection | *I think this test is very simple and easy to do. It is better to do it to take care of your own health. Moreover, this test is free. This is something good for us because we are allowed to do the test.* ***(Chinese/M)***  *I agree to complete the test. It’s better to know early if we have any symptoms, probably we could be saved before it’s too late.* ***(Indigenous /M)***  *This is one of the ways to protect ourselves from this cancer. If we do the test, it would be helpful to get to know the risk for the cancer.* ***(Indian/F)***  *If it’s an internal illness, which means we can’t see with the naked eye; it’s better to do a test. Once you know, you become more careful of yourself.* ***(Malay/F)***  *When doing the test, the doctor may advise on diet and lifestyle factors that could help us to prevent colorectal cancer. I am sure the doctor will give appropriate advice for us when we meet to do the test.* ***(Indian/M)*** |
| Barriers towards CRC screening | Lack of doctor’s recommendation to complete iFOBT | *I'm hearing [about] this test for the first time here. Maybe, only those who have bowel problems will know and so far, the doctor did not talk about it.* ***(Malay/F)***  *Yeah, at clinics we are just doing general tests like urine and blood tests only. There was no opportunity to do a cancer screening test like this.* ***(Indian/M)***  *It is because if the doctor did not mention about iFOBT, we will not get to know about it.* ***(Chinese/F)*** |
|  | Service barriers   - Waiting times at clinics - Transportation | *I'd say here in Bekok health clinic if you want to do anything like that, it's not a half-hour wait. It's 2-3 hours of waiting. That place is small. Even to pick up medication for fever, it takes 1-2 hours of waiting, waiting for your turn.* ***(Indigenous /M)***  *If there is a little pain, then I will go to the government clinic as it takes time to see the doctor. If in case of severe pain, I will immediately go to the private clinic as there is less waiting time and can see the doctor as soon as possible. In government hospitals we have to wait to follow the colour zone like green, red and yellow. So, depending on the severity of the condition, I have to plan where to go.* ***(Indian/M)***  *Some can't go to the clinic because they have to wait for their kids to send and all that. Though it's 3km, but not all can go to the health clinic.* ***(Malay/M)***  *There should be someone who comes and takes it. I don’t have the time if I had to go and come back from the clinic. Right now, the issues would be motorcycle problems, the rainy season.* ***(Indigenous /M)*** |
|  | Financial concerns   - Cost of treatment and medications at private hospital/clinics | *The treatment will require costs, which we cannot afford to.* ***(Malay/M)***  *I also worry about money when doing the colonoscopy.* ***(Chinese/M)***  *Better go to the government hospital, you don’t need to pay. We have to use the facilities that are available.* ***(Malay/F)***  *Yes, I do feel it a little but it’s not that deep worry, just the normal worry. It’s normal for poor people like us. Ahh. It’s only the financial issues, that’s about it.* ***(Indigenous /M)***  *Nowadays, the hospital doesn't even have a free one, senior citizens will not get it free too.* ***(Chinese/F)***  *It is better for me to go to the government hospital as financially it is not good to go to a private hospital.* ***(Indian/F)*** |
|  | Emotional barriers   - Embarrassment - Disgust | *The problem is that it feels disgusting, that's all. That's the problem.* ***(Malay/F)***  *Feel like no manners by taking the stool to the doctor. I won't do it. Just feel like not to do it la.* ***(Chinese/F)*** |
|  | Concerns about postal intervention   - Unreliability of postal services - Distrust - Illiteracy and language barrier | *Because our postal service is slow.* ***(Malay/M)***  *Sometimes, the postman will get it to the wrong place.* ***(Chinese/F)***  *I might be wondering what this is? When reading the letter, I might feel like why I need to do it if I have no symptoms or anything related to that. I feel sending a kit via post would not work because Chaah is not an urban area. So I don't think people would be aware of this when sending via post.* ***(Indian/M)***  *I wouldn’t and probably will ignore it. I don’t provide any of my details without knowing it.* ***(Indigenous /M)***  *I absolutely wouldn't trust it. I want to know if it’s for real or not. I will visit the doctor and ask them; I’ll bring everything.* ***(Malay/F)***  *I have to think and figure out about the source of the parcel.* ***(Indian/F)***  *We can’t really read in Malay. Even in Tamil, if we read and could not understand then it would be difficult. Those who can’t read well would be tough to understand the leaflet.* ***(Indian/F)***  *Just don’t have it in English, I'm fine.* ***(Indigenous /F)***  *It would be best to have pictures and 3 languages. If you sent it in English we would not understand, Malay is still a little bit more understandable.* ***(Chinese/M)*** |
| Suggested strategies to enhance iFOBT uptake/ completion | Collaboration with community support groups and non-governmental organisations (NGOs) | *It's through a committee that has people they are familiar with like KOSPEN and we will create a committee that will execute this programme.* ***(Malay/M)***  *Organisation like Lion Club and KRT also have organised cancer screening tests such mammogram. Everyone will come.* ***(Chinese/F)***  *It will be very helpful if SEACO could arrange a screening test for us. It will be better to get tested and avoid anything in beginning stage itself.* ***(Indigenous /M)***  *I personally think that we have given enough support and facilities for to the community. As members of the KRT committee here, we used to organise a lot of health screening activities and try to fix most of the issues among people here specifically for the access to health services. So, I think if you could approach KRT committee members to spread and help out for this project, it would not be a problem.* ***(Indian/M)*** |
|  | Collect stool container from clinics | *It is better, to go the clinic as it is convenient for us. It is because if you need someone to post it, some of the people may not know how, so by taking it to the clinic everyone knows.* ***(Chinese/M)***  *I think the best way to receive the kit is by collecting from the clinic. This is because we used to go to the clinic regularly. So much easier for us to ask the nurse and get the kit.* ***(Indian/M)***  *The best is the nearest one which is the clinic. I would agree with that. By invitation we visited a clinic to get the test kit. It’s hard to gather everyone to participate in any event.* ***(Indigenous /M)***  *I prefer to receive it from the clinic. We used to go to the clinic, and we have the record as well. (****Indian/F)***  *The best if it’s at the clinic. We can collect the kit from the clinic.* ***(Malay/F)*** |
|  | iFOBT invitation letters and information leaflets | *In my opinion, it is better to put up a banner saying that you will receive a letter in advance. This is because most of our people are not well educated, so it is better to include messages in 3 languages, Malay, Tamil and Chinese. Then when they receive the letter from a government clinic or hospital, they will be serious about it and will take necessary action.* ***(Indian/M)***  *Sometimes, I would not be bothered if I'm not told in advance.* ***(Indigenous /F)***  *But if received via post will be fine for those who can read, but for those who can't read, will be difficult as well.* ***(Indian/F)***  *It would be best to have pictures and 3 languages. If you sent it in English we would not understand, Malay is still a little bit more understandable.* ***(Chinese/M)*** |
|  | Returning stool container | *Yes, we will return the specimen bottle to the clinic.* ***(Indian/F)***  *Ahh, there's no problem in sending it back to the clinic.* ***(Malay/F)***  *On my own, I send it on my own.* ***(Indigenous /F)***  *You can’t expect the clinic to pick it up for you, right? We have to send it back ourselves.* ***(Indigenous /M)*** |
|  | Reminders | *Older people especially, wouldn’t be able to remember about this, so need reminder like calling them.* ***(Indigenous /M)***  *Remind us, that means they have our phone number; so, they call us back. Yes. If you want to get 100% results, that needs a reminder.* ***(Malay/M)***  *Yes, we should receive a reminder. I agree to have a phone call and so on. I think the clinic staff can check based on the list and give the reminder calls.* ***(Indian/M)***  *Reminder would be better by phone call.* ***(Indigenous /F)***  *Some people tend to forget, so it is good to call and remind them. Older people especially, would not be able to remember about this.* ***(Indian/F)***  *Ahh. Because there's already a message; so, I won’t ignore it. I would then take it seriously.* ***(Malay/F)***  *It is not necessary for reminders. It is our responsibility to do it once received.* ***(Chinese/M)***  *No need la, we will be responsible for doing it ourselves. If there is reminder, then you will feel like more pressure, if not then maybe you will wait and wait for tomorrow and then the day after tomorrow.* ***(Chinese/F)*** |
|  | Receiving results | *If we can get the results from the doctor, it would be easier for us to get consultation.* ***(Indian/F)***  *Get the clinic to call the respondents who have this kind of case. No need to call those who are fine. They give a timeframe. If after a month, there's no call; that means you're fine.* ***(Malay/M)***  *By mailing it, we can take this black and white to whichever doctor, right? Then, we can know by showing the doctor our results and asking them to interpret it.* ***(Malay/F)*** |
|  | Health and screening events | *I think should do a big campaign promoting awareness on this colorectal cancer and stool test. If you do before deliver the kit, then it will be fine.* ***(Indian/M)***  *Having a campaign and a talk would be good enough.* ***(Indigenous /M)***  *I went to this medical camp organised by the Hindu Sangam. There, I did cancer screening tests including this colon cancer. For example, like taking a stool sample and they said no problem. I did about 1 to 2 months ago.* ***(Indian/M)*** |
|  | Incentives | *[…] when you want to organise such events, you should provide a hamper to the participants. Just to attract them to the event. It's difficult to make them participate without anything in return.* ***(Indigenous /M)***  *For example, you put an ‘x’ amount per trip, RM20. Perhaps, I think many will go. Transport cost is a burden.* ***(Malay/M)*** |
