# Supplementary material for: Facilitators and barriers to colorectal cancer screening using the immunochemical faecal occult blood test among an average-risk population in semi-rural Malaysia: A qualitative study
Source: PLoS One. 2022 Dec 29;17(12):e0279489. doi: 10.1371/journal.pone.0279489 (PMC9799312; doi:10.1371/journal.pone.0279489)
Supplement: Attachment — Submitted filename: PLOS Qualitative barriers to bowel screening.docx [file pone.0279489.s004.docx]

Thank you for inviting me to review this manuscript.

The authors have reported on a topic which is highly relevant and important for bowel cancer control. This qualitative study has uncovered several critical issues that may require further attention in order to encourage uptake of bowel screening amongst the Malaysian population in the future.

There are several areas in the manuscript which require further clarification:

1. Sampling

- Purposive sampling was conducted for this study and this was aided by a community organization called SEACO. Some inclusion criteria were mentioned but were there exclusion criteria – would prior personal experience of bowel screening affect recruitment into the study. What measures were taken to minimise sampling bias?

- The cohort of individuals recruited into the study are primarily from a rural community and where most of the interviewees’ educational level are up to secondary education. I feel the word rural community needed to be included in the title as these findings may not be truly representative of Malaysia as a whole nation. This should be mentioned in the limitation’s chapter.

2. Methodology

- During the FGD, were attempts made to reduce bias from dominance effect, halo effect and group thinking?

-	The data analysis was described in phases but could do with further elaboration. The authors used an iterative approach but what method was used to move from codes to themes (e.g. inductive? Deductive analysis?).

- There was no mention of attempts made to determine whether data saturation was achieved?

3. Results

- The results section was mainly descriptive and significant sections lacked in-depth analysis. For example lines 208 -228: Cancer awareness theme, section of Barriers to CRC screening. Statements were made to summarise the included quotes but there was limited analysis of how these subthemes may relate and influence decisions to screening. The theme on Barriers to CRC screening is also rather short – particularly on stool collection - this is probably one of the most important subthemes that has been uncovered and warrants further elaboration and specifics.

- There were statements which did not fully reflect the included quotes e.g. line 247. The statement about participants’ perception of self susceptibility to CRC is not supported by the included transcript.

- Under Theme 5 – suggested strategies to improve iFOBT uptake or completion, are the authors implying that certain strategies are more likely to be suited for specific ethnic groups as several statements have attempted to draw associations with certain ethnic groups?

4. Conclusion

Line 39 – ‘Another barrier that people faced was financial constraints’.

Whilst I can see what the authors are alluding to, the data could also be interpretated as bowel screening is affordable to everyone through the public health system in Malaysia, but this is limited by capacity – long waiting times etc. An alternative would be to seek private health care screening facilities, but this would incur costs to the patient and would thus limit access to those who cannot afford it. I would therefore recommend clarifying the first sentence to this paragraph.

5. Others

- Is there a typo on Line 106 – 3^rd^ section mentioned but 2^nd^ section was not mentioned

- Typo on Line 180? – ‘stated that they would *not* ask for other preventative health screenning’ – is this true given the subtheme here is about participants reporting that they would actively ask their doctor about cancer screening

- Line 358 – missing or incomplete sentence
